# Re-Examining Labels in Neurocognitive Research: Evidence from Bilingualism and Autism as Spectrum-Trait Cases

**DOI:** 10.3390/brainsci12081113

**Published:** 2022-08-22

**Authors:** Maria Andreou, Vasileia Skrimpa

**Affiliations:** 1 Department of Speech and Language Therapy, University of Peloponnese, 24100 Kalamata, Greece; 2 Department of English, School of Arts and Humanities, University of Cologne, 50931 Cologne, Germany

**Keywords:** labelling, individual variation, bilingual children, children with autism

## Abstract

Despite the fact that the urge to investigate bilingualism and neurodevelopmental disorders as continuous indices rather than categorical ones has been well-voiced among researchers with respect to research methodological approaches, in the recent literature, when it comes to examining language, cognitive skills and neurodivergent characteristics, it is still the case that the most prevalent view is the categorisation of adults or children into groups. In other words, there is a categorisation of individuals, e.g., monolingual vs. bilingual children or children with typical and atypical/non-typical/non-neurotypical development. We believe that this labelling is responsible for the conflicting results that we often come across in studies. The aim of this review is to bring to the surface the importance of individual differences through the study of relevant articles conducted in bilingual children and children with autism, who are ideal for this study. We concur with researchers who already do so, and we further suggest moving away from labels and instead shift towards the view that not everything is either white or black. We provide suggestions as to how this shift could be implemented in research, while mostly aiming at starting a discourse rather than offering a definite path.

## 1. Introduction

The concept of heterogeneity among cognitive and neurodevelopmental characteristics across participants in research studies and the significance of individual variation has been gaining ground in research methodology in a variety of fields, making it more evident that the consideration and further integration of individual variation in methodological strategies follow the current trend in the clinical and research field. Nevertheless, despite the significance given in literature on taking into account the variability across populations, research investigating cognitive systems, such as language, or neurodegenerative disorders, autism being one of them, still tends to categorise individuals into groups that can be attributed with a specific set of characteristics and traits. The process of grouping individuals (adults or children) to fit under certain research criteria results in the creation of labels towards an arbitrary stratification which, in the majority of cases, does not take into consideration individual variation.

Cognitive and neurodevelopmental trajectories in children demonstrate similar patterns; however, the deviations in research findings and the lack of replicability in results often stem from the absence of proper definitions of the systems under investigation, as well as generalisations of the findings across populations. In the case of spectrum-trait systems or conditions, such as bilingualism and autism, the absence of clarity in the criteria distinguishing bilinguals from monolinguals or neurotypical individuals from atypicals is evident [1,2].

Bilingualism refers to language as a cognitive system. It deals with the acquisition of two language sets and is viewed as a continuum [3], encompassing a set of individual and context-related characteristics, such as dominance, age of onset, language history and status of language use [4,5]. However, across the literature, bilingualism has been investigated in terms of binary definitions and categorical labels that arbitrarily distinguish monolinguals from bilinguals, without considering the variability in characteristics, social implications or level of bilingual experience [6], leading to mixed research outcomes that do not exhibit sufficient replicability.

Autism on the other hand is labelled as a spectrum disorder that entails a variety of diagnostic criteria. It is a fact that the idea of severity of the symptomatology as well as individual variation in clinical populations is highly relevant in terms of clinical research studies. Nevertheless, outside of strictly clinical settings, it is often the case that in an attempt to achieve unanimity in terms of symptomatology and diagnostics, the large heterogeneity that this neurodevelopmental condition demonstrates, as well as the individual differences among autistic individuals, is often examined in a monolithic manner that is usually manifested through inadequate sample sizes in research study participants and generalisations of the findings across large autistic populations [2,7]. Conceptualising and comprehending the large individual variability in autism, as well as the factors influencing its manifestations might constitute a challenge, should however be approached in a multidimensional analytical way.

We propose that the problems that are evident in research with respect to the lack of consensus in the findings, inaccurate generalisations and absence of replicability and credibility stem from inconsistencies in the stratification and insufficient group labelling. The present paper questions the efficacy of the classification of research participants into groups that systematically ignore individual differences among individual participants and treat spectrum-trait cognitive and neurodevelopmental cases, such as bilingualism or autism, as monolithic variables. A more in-depth description of the selected spectrum-trait cases under investigation in the present paper—namely bilingualism and autism—will assist in shedding light on the specifics of each case and facilitate understanding over the operations as well as limitations that further promote the need for a change of course in research methodological strategies followed in both bilingualism and autism.

## 2. Individual Variation in Bilingualism

### 2.1. General Overview of Bilingualism

A wealth of studies have investigated how the experience of being bilingual shapes our language and cognitive abilities. In terms of their language abilities, bilingual children seem to have smaller vocabularies compared to their monolingual peers [8], and their grammatical abilities may differ from those of monolingual children, depending on the grammatical structure tested [9]. In terms of their cognitive abilities, there is conflicting evidence about whether or not bilingualism leads to advantages in executive functions (EFs), i.e., the cognitive processes responsible for goal-oriented behaviour, the capacity to think ahead, suppress impulses and temporarily hold information [10].

However, is this always true? There are studies that fail to replicate the abovementioned results and for instance do not find advantages in the cognitive abilities of bilingual children [11]. Most studies testing for bilingual advantages use measures and tasks that do not have demonstrated convergent validity, and any significant differences in performance may reflect task-specific mechanism and not domain-free executive function (EF) abilities. The cumulative effect of confirmation biases and common research practices has either created a belief in a phenomenon that does not exist or has inflated the frequency and effect size of a genuine phenomenon that is likely to emerge only infrequently and in restricted and undetermined circumstances. To present alternative justification with respect to the non-replicable and confronting results, we will present two studies that investigated the bilingual advantage that, despite using categorical variables, are massively influential, as reflected by citation counts ([12], over 425 citations; [13], over 550), but that did not objectively measure bilingual language status. In the study by Carlson and Meltzoff [12], 50 native bilingual, late bilingual and monolingual kindergartners were administered a battery of nine cognitive control tasks and an English proficiency test. The three groups had statistically indistinguishable scores in all nine cognitive control measures, and the bilingual children had lower English proficiency scores. The latter result is hardly surprising: after all, these children spent half their waking hours speaking Spanish. However, the authors then went on to control for differences with respect to English proficiency and socio-economic status (SES). When they did, the bilinguals showed higher scores in three out of the total of nine cognitive control tasks, as well as a composite measure of cognitive control. These results were interpreted as consistent with the bilingual advantage hypothesis as evidence that bilingual children can do more intellectual work with fewer available resources. However, the authors in this case only controlled for specific variables that boost the bilingual advantage, without considering others as well. Despite the fact that there was some evidence of a bilingual advantage in the way the data were statistically interpreted, it would have been more scientifically preferable to create a continuous variable that would take into account all variables that constitute differences among the groups (i.e., vocabulary knowledge in both languages for the bilingual children, SES, demographic information, parental input, etc.).

The second influential set of findings comes from a study by Bialystok [13], in which 60 Chinese-English bilinguals and English monolinguals (all from Toronto, ON, Canada) were administered a pair of cognitive control tasks and an English proficiency test. Bilinguals showed higher scores than monolinguals in both cognitive control tasks. As was the case with the Carlson and Meltzoff study, these results were interpreted as consistent with the bilingual advantage hypothesis. While this may well be true, it is equally plausible that these findings were driven by uncontrolled confounding factors endemic to between-subject designs, and there are a number of clues pointing to this possibility. First, despite the fact that the monolinguals in this study had about twice as much experience speaking English as the bilinguals, they were no more proficient in English than the bilinguals. Second, monolinguals not only struggled in the challenging component of the cognitive control tasks, most notably the post-switch trials of the Dimensional Change Card Sort (DCCS) task, they also struggled to remember what they were asked to do in the DCCS task. Beyond this, Asian cultures place a premium on self-regulation, and in many Canadian cities, enjoy socioeconomic advantage. Thus, there are reasonable grounds to doubt that the superior performance of the bilinguals was attributable to the effects of language status alone. It is also worth pointing out that both studies claimed to be studying the effects of bilingual language status, but neither measured the proficiency of bilinguals in their two languages.

### 2.2. The Role of Individual Variation in Bilingualism

One common denominator for studies on bilinguals is the individual variability in the participants’ performance on language, cognitive abilities and language processing tasks. We believe that individual variation is responsible for the conflicting results. Driven by this belief, we will present studies that highlight the importance of individual variation.

Pliatsikas et al. [14] suggested that stereotypical “bilingual versus monolingual” comparisons should be avoided. The authors argue that recently the attention of the field has drifted toward more explicit techniques, that take into consideration the individual bilingual experiences, as indicated in neuroimaging and behavioural research studies. These findings argue whether the perspective of the bilingual experience would be a significant factor for the interpretation of the deviation of the research outcomes, and whether the focus should be placed on methods that take these experiences into consideration. They conclude that the need for elimination of binary bilingual/monolingual relations has been suggested by various studies, posing new methodological suggestions that place emphasis on the individual variation of bilinguals. Such methodological examples include two particular approaches followed towards the elimination of the binary categorisation of monolingualism versus bilingualism, since in most cases they are conducted without the presence of a monolingual control group. One of the approaches corresponds to the implementation of bidirectional multimodal schemes that investigate the individual variation in language production of bilinguals in terms of duration, frequency, proficiency, age of onset and exposure targeted at outlining the level of neurofunctional adjustment. Another approach the authors mention is the conduction of longitudinal research studies in the case of bilingual populations that aim at examining individual trajectories of bilingual language use, language exposure and cerebral adaptation in non-structured experimental conditions throughout a specific period of time. These approaches take individual variation among bilingual populations into account avoiding resorting to monolithic labelling and distinction among bilingual and monolingual individuals.

De Bruin [15] also highlights the importance of individual variation. She suggests that in order to facilitate a more accurate comparison among research studies, it is crucial for researchers to describe information with respect to the onset of acquisition and mastering of the languages, as well as with respect to the way bilinguals acquired those languages and the general social and educational setting. She argued that researchers so far prefer a categorical model by assessing similar and different patterns between early and late bilinguals. In her article, she suggested that considering bilingualism as a continuous variable facilitates the perception of bilingualism as a spectrum and could account for the variability among bilinguals.

Kremin and Byers-Heinlein [16] suggest in their study that the domain of bilingualism can benefit from other psychological domains by incorporating progressive psychometric designs, which integrate categorical and continuous features. They suggest that these designs can merge division among monolingual and bilingual groups taking into account recent suggestions, according to which bilingualism should be considered a continuous variable. Analyses can therefore be done based either on continuous or on categorical information, or on both types of information, with respect to its validity regarding the aim of their study.

As we can see from the above studies, it is obvious that more and more researchers are concerned about the existing labelling in the field and propose re-examinations. As already mentioned before, the idea that bilingualism should be considered a continuum, instead of discriminating between monolingual and bilingual groups, is gaining more ground [15,17,18,19]. This suggestion has a significant impact on the way bilingualism should be theoretically perceived and on the data analysis methods (Table 1).

## 3. Individual Variation in Autism

### 3.1. General Overview of Autism

Another area that has fertile ground for the study of individual differences is that of neurocognitive disorders. In particular, for the purposes of this study, we will focus on children with autism. Autism Spectrum Disorder (ASD) is a neurodevelopmental disorder that demonstrates big phenotypic heterogeneity [20] and big deviations in individual level. According to the common definitions, the umbrella term of ASD describes a wide range of conditions that exhibit common characteristics, such as deficits in social and communication skills, limited interests and repetitive patterns of behaviour. The term has emerged to replace the until recently widely used term “Pervasive Developmental Disorders” [21], which described a number of congenital disorders, such as Autistic Disorder, Rett Syndrome, Childhood Disintegrative Disorder, Asperger’s Syndrome and Pervasive Developmental Disorder not otherwise specified (PDD-NOS) [22]. It is important to highlight the fact that in spite of the common characteristics, ASD presents big deviations in individual level, hence it is described as a spectrum, a term used in the diagnostic guideline of the condition for placing emphasis on the heterogeneity with respect to the severity of symptomatology and general abilities [22].

The variation presented by the sub-categories of the “Pervasive developmental disorders” was one of the starting points for discrimination among symptomatology and placing emphasis on the individual differences. For instance, Asperger’s Syndrome described individuals with autism that had nevertheless good cognitive functions and almost no language impairments. The language use, however, of these individuals was different to their neurotypical peers [23]. On the other hand, “Pervasive Developmental Disorder not otherwise specified” referred to individuals that combined many different autistic symptoms. The recent revised version of the American Psychiatric Society’s DSM-5 system [24] has removed Asperger’s Syndrome and Pervasive Developmental Disorder not otherwise identified, while at the same time moving Rett Syndrome and Childhood Disintegrative Disorder to other diagnostic categories. At the same time, it distinguishes ASD at three levels of severity, depending on the degree of support the individual with the disorder needs.

The aetiology of ASD contributes to variability and should therefore be considered. The emergence of ASD is pathogenic; nevertheless, it remains unknown. Modern research attributes the pathogenesis of ASD to epigenetic causes, i.e., a combination of inherited genetic predisposition and environmental conditions that may adversely affect the genome. Furthermore, ASD may coexist with attention deficit disorder, with learning impairments, communication deficits, as well as psychoneurological conditions [24]. There is no evidence in the literature that psychological factors can account for the causes of ASD, which occurs four to five times more often in males than in females, and also more often in siblings of children that already have the disorder than in the general population.

Over the last 30 years, the emergence of new confirmed cases of individuals with ASD has been increased significantly, mostly due to the more standardised diagnostic methodology used, distinguishing autism from other neurodevelopmental or psychiatric conditions when the criteria of its symptomatology are met [25]. However, when individual variation is not systematically taken into account during the process of providing diagnostic classification of a disorder that demonstrates such variability and combines intellectual and developmental factors as well as level of severity of the symptoms, it could not only lead to discrimination, but could also make no positive contribution towards educational and therapeutic practices [26].

### 3.2. The Role of Individual Variation in ASD

Heterogeneity in ASD has been approached across a variety of levels of analysis that include cognitive factors [27], neurodevelopment [28], genotypes, behavioural patterns and language development [29], as well as factors that correspond to research approaches to the diagnostics of ASD in terms of similarities vs. differences across the autistic populations, dimensional or categorical distinctions of diagnostic criteria and outcomes with respect to intervention or treatment [30,31].

It should be taken into consideration that the interpretation of the term “spectrum” itself and its reflection on research outcomes demonstrates deviations. On the one hand, the term could attempt to describe the heterogeneity within autism as a disorder and the gradual increase in severity level of its symptomatology in multiple levels of analysis. On the other hand, “spectrum” can also refer to a more qualitative distinction among individuals, ranging from no evidence of autistic traits to a more evident diagnostic need, taking into account the whole population and adopting a more inclusive stratification [32]. It is, however, evident that individual variation plays a significant role in ASD research, not only with respect to social-communication differences among individuals within the spectrum, but also due to the different response of these individuals to medical therapeutic approaches and behavioural interventions [33].

Another issue that should be taken into consideration with respect to individual variation within the spectrum is the fact of non-replicable research findings. It is often the case that individuals with autistic traits are given a diagnosis based on those criteria that best fit their symptomatology, and, although comorbidity of other disorders is taken into consideration, it is the performance on specific tasks that determines their diagnostic status, which might not reflect impairments in social, intellectual and learning abilities [34]. On the other hand, the large heterogeneity within the spectrum seem to influence the outcome of research studies, when not being taken into account. It is indicated in the literature that data deriving from autism research studies with respect to neurodevelopmental, cognitive and language factors do not show significant ASD effects [35], due to the lack of standardised diagnosis of the big variability of the symptomatology, as well as poor categorisation of the autistic criteria in terms of individual factors such as severity of the condition, age and intellectual level [36].

Waterhouse and Gillberg [7] critically discuss the attempt of researchers to unify the symptomatology of autism under one sole brain dysfunction, pursuing a legitimate neurophysiological explanation for the disorder (Table 2). This attempt, nevertheless, has resulted in neglecting individual characteristics of different people diagnosed with autism and has further promoted the lack of replicability of research results that could not be applied across the spectrum. The authors suggest three major changes that research in the field of autism has to take into account. More specifically, they reject the notion of one single unifying diagnosis for autism that can be restrained under one neurobiological deficit. In that sense, they also criticise research studies that perform arbitrary observations by dividing groups into “controls” and “autistics”, without considering individual differences. Their second point concerns the fact that each single autistic trait might be a concurrence of a variety of cerebral impairments and, in that respect, every impairment in the brain could manifest through different traits [37]. The authors present the suggestions of scientific studies for dealing with the issue and make individual differences regarding biological sex, symptomatology, cognitive and intellectual abilities, as well as comorbid conditions more prominent [38]. Finally, the third point of change the authors suggest addresses the issue of data analysis methodology towards a more inclusive model with regard to individual variation, especially in the case of brain functions.

Uljarević et al. [39] reported the significance of individual variation in ASD research with respect to sensory features. They bring up the issue of refraining from conducting group comparisons classifying participants under unifying labels but rather emphasise the subtypes that differentiate the autistic symptoms and their neuropathological origins. The authors propose that what should be taken into consideration are the more fine-grained multidimensional and longitudinal approaches in autism research that focus on heterogeneity in terms of individual variation from an early age that has an impact on the holistic clinical image of individuals within the spectrum.

In their review, Happé and Frith [40] also highlight the implication of heterogeneity and individual variation in autism with respect to changes that research in ASD has witnessed, as well as their impact on future methodological and interventional approaches. More specifically, the authors point out seven significant differences in the concept of autism, namely a more broad variety of diagnostic autistic traits, a general acknowledgment of the frequency of occurrence of autism across populations, the lifelong character of the disorder, a condition that entails multidimensional aspects, acceptance of individual variability and transition from a holistic diagnostical approach to the concept of “many autisms”, acceptance of comorbid condition occurrence and finally the approach of neurodiversity in autism. Regarding individual variation, Happé and Frith point out that the underpinnings of the condition are multifaceted, which further contributes to its complexity and dimensionality. More specifically, research distances itself from approaches of “autistic” versus “non autistic” categorisations and rather focuses on markers that prognosticate individual variation, considering a.o. genotypic, environmental and behavioural parameters.

A study by Silleresi et al. [41] broadened the investigation across the total of the autism spectrum targeting the intellectual and structural language skills of children, with a particular emphasis on individual profiling. They administered a set of tests, such as nonword and sentence repetition tasks, as well as tasks for testing cognitive skills to test the language and cognitive abilities of 51 children who were 6–12 years of age. Their results indicated the existence of five profiles (instead of four, as suggested by ISD-11) among the autistic individuals with respect to language deficits. More specifically, on the one hand, intact language abilities emerge in combination with deficits in non-verbal intelligence, and on the other hand, intact non-verbal intelligence is presented along with language deficits. The findings of this research contribute to the development of specific interventions that fit to individual profiles and particular impairments.

To conclude, we should raise awareness to a very important issue that should be addressed with respect to diagnostic generalisations and research replicability issues, which is the fact that the criteria for the ASD diagnosis occur from data collected from patients in Western countries. It has been recently suggested that, apart from neurophysiological factors, what can also have an influence on diagnostic outcomes is the extent to which the diagnostic tools developed are socially acceptable in other countries [42], as well as the extent to which a combination of symptoms would lead to a diagnosis of ASD or to another neurodevelopmental disorder. For instance, cross-cultural conditions may contribute to the heterogeneity of the spectrum, depending on which diagnostic methodology they use or reject (i.e., Asian cultures reject eye contact as a criterion, as they consider eye contact disrespectful [42]. It is also the case that current research is not based on individual diagnostics with regard to ASD, but rather on a plethora of data that discriminate between typical and atypical populations, in combination with the fact that the majority use cataphoric rather than anaphoric diagnostic methodology, resulting in the production of a big body of findings without replicability value [43]. This issue should be taken into consideration when proposing suggestions for methodological improvements and better integration of external variables that contribute to heterogeneity, such as demographic information, socioeconomic status, diagnostic opportunities, etc.

## 4. Plausibility of Bypassing Labelling

All the above findings lead us to the view that the labelling of groups is absolute (bilingual vs. monolingual, typical development vs. non-typical development). It is evident that a new approach needs to be followed by taking into consideration the importance of individual variation. However, the plausibility of the total elimination of labelling, especially in terms of clinical research, should also be taken into serious consideration. This chapter will review selected suggestions that have been recommended in the literature, as to whether and how that shift could be implemented.

Taking into account evidence from the literature, one path that has been suggested as a strategy to bypass labelling is that of neurodiversity. With respect to the classification of ASD as a neurodevelopmental disorder that encompasses a variety of deficits at the social, communication and interpersonal level, the literature suggests an approach that does not focus on limitations and disabilities that raise the urge for intervention [44] but rather to shift the focus on the diversity of autism in terms of different dimension of perception, processing, comprehension and experience [45]. The perspective of disability that has been attributed to autism and contradicts the traditional “neurotypical” social expectations has been viewed as obsolete. More specifically, the idea of the neurodiversity of autism as a movement towards inclusion and elimination of discrimination on the basis of impairments has started gaining traction in research methodology [46]. The movement advocates that a variety of conditions that demonstrate neurocognitive variations from the neurotypical populations should be treated as dimensions of human diversity and the processing of sensory information as another possible alternative [45].

Research presents a general notion of narrowing down neurodevelopmental conditions into their symptomatology, attributing disadvantages and susceptibility that further result in social discrimination, isolation and lack of proper integration. Biological diversity is adopted as a natural variation across the function of the brain by the notion of neurodiversity, which rejects the processes of labelling as “impaired” or “degenerated” [47]. Comparisons among “typical” and “atypical” populations stem from the collective idea that cognitive and behavioural differences can only be interpreted as deficits of the “ideal” neurocognitive type that demonstrate a specific set of processing skills and patterns, regarding deviations as non-ecological, and therefore in need of intervention [48]. However, it is crucial to consider that social and cultural expectations shape the notion of neurotypical classification. Neurological diversity among humans is associated with anatomical differences in the brain and synaptic properties which can be perceived as the difference in sensory experience and processing of information rather than deficits [47]. More specifically, it has become more evident that referring to disabilities when researching autism corresponds more to the perception of social norms as the set of characteristics of an individual that fit the description of their social context, whereas it is the failure of this social context to accommodate the individual needs of these individuals [49].

Lombardo et al. [2] also underline the big heterogeneity across ASD, and they argue that the problems stem from labelling individuals within the spectrum by placing them, on the one hand, under categories that fit the diagnostic criteria but ignoring, on the other hand, their individual differences. The authors criticise research methodologies that include a small number of participants and draw generalised conclusions. They rather suggest the significance of big data in disentangling the big variation that autism demonstrates, and they emphasise the broad and deep character of this approach in terms of sample sizes and levels of data analysis to promote comparability and replicability of research findings in the field of autism that is inclusive of individual variation among autistic populations. In their paper, the authors provide valuable details about computational models that examine and analyse the heterogenic patterns of autism and the directionality of which could be either bottom-up or bottom-down, taking into account the developmental trajectories of individuals in the spectrum. Finally, the authors tackle the value of integrating “unsupervised” machine learning models into heterogeneity-based research in autism, which does not require an a priori knowledge of autistic traits and symptomatology, but rather depends on the divergence that is encompassed within the data themselves. The “unsupervised” model could accommodate a more inclusive approach in terms of individual differences and a less biased methodological design.

Taking into account individual variation with respect to the particular traits of individuals within the spectrum can shed light on the fluctuation in the performance in cognitive, linguistic, social and behavioural tasks, which can result in a lack of replicability of scientific findings. It is also crucial to consider that applying dichotomic practices in terms of terminology and labelling in ASD (“low functioning” vs. “high functioning”) sets biases towards lower expectations and promote discrimination that presupposes a lack of competence and inability of positive achievement and success [50]. Investigating autism according to the paradigm of neurodiversity broadens the perspectives of developing scientific methods towards the elimination of research errors by respecting diversity as a type of natural human variation [51], without implying, however, that individuals with ASD do not require support for social integration, but rather focus on practices that do not include reduction of the autistic traits [33].

Another proposition towards the gradual elimination of categorical labelling is a re-examination of the existing terminology and definition of bilingualism and autism, targeting a more precise and inclusive approach. The literature review by Marian and Hayakawa [52] underlined the big heterogeneity bilingualism presents as a phenomenon and gave emphasis to the importance of the emergence of a methodological approach that would bring consensus as to what bilingualism entails and how it should be approached when conducting research. The authors provide suggestions for future steps that are worth mentioning in this review. Most importantly, they propose the creation and validity of a bilingualism quotient (BQ), in a similar manner to that of the intelligence quotient (IQ). For operationalising bilingualism, they suggest the inclusion of correlational variables to BQ, such as manner of language acquisition, language proficiency, age of onset, language identity and language switching. Despite the fact that this suggestion does not come without limitations (one could argue that scoring the skills of an individual could be considered equal to labelling), it is a proposition directed towards the creation of continuous indices that entail significant variables. At that point, they also stress the urge for a more fine-grained definition of bilingualism as well as which individuals could be called bilinguals. In that scope, the authors further recommend the establishment of a general consensus among scientists and researchers in the field that could promote a more unified approach to the continuum of bilingualism, which would also include evaluations of over-time alterations, larger datasets with more variability and standards that would take into account demographic information. Last but not least, they recommend the creation of measuring tools that are easily accessible, as well as applications for data acquisition and retrieval. Finally, they put emphasis on the establishment of better communication among researchers towards a more holistic understanding of bilingualism.

One more study that adopts the approach of consensus for bilingual terminology and proximity of research methodology and assessment instruments towards the elimination of arbitrary comparisons between monolinguals and bilinguals, is that of Kašćelan et al. [53]. They discuss the validity of different types of questionnaires for evaluating bilingual experience, as well as to what extent these questionnaires could be comparable to one-another. The authors analysed around 50 questionnaires administered in a number of research studies along with the assessing intentions underlying them. The results reveal great deviation in the operationalisation of the bilingual components, namely language use, language proficiency and language exposure in these questionnaires. The review calls attention to the urge for transparency of the reporting of bilingual research findings and recommends a general collaborative agreement among scientists with respect to their methodology and tools as future steps towards research comparability and validity.

The issue of heterogeneity in both bilingualism and autism was also recently raised by the work of Prévost and Tuller [54], that critically investigated research outcomes with respect to bilingual language development in children with autism in an attempt to shed light on the complexities of each state, (namely bilingualism and autism) by cross examining the two. The authors put emphasis on precision when it comes to profiling bilingual children in the spectrum, taking into account the large variability inherent in trajectories of language acquisition and development, that will have future application on more targeted language interventions and development of tools modified to fit the requirements of bilingual and autism research. They promote the need of a more fine-grained definition of bilingualism and autism that will facilitate a deeper comprehension of these experiences, as well as the interplay of them both. Variables such as age of onset, proficiency, level of exposure and linguistic proximity of the two languages are suggested to be taken into consideration for better approaching bilingualism on the one hand [55], and on the other, impairments in language acquisition or intellectual and learning deficits that should be considered when investigating individuals with autism.

Prévost and Tuller’s [54] work constituted the starting point for a fruitful discussion concerning the problem of replicability of research results that in the most part fail to take into account individual variation of bilingual individuals and individuals with autism, as well as the extent to which those results are further applicable. Digard and Sorace [56] argue that methodological strategies should drift away from categorical measures for bilingualism and autism that focus on non-adequate language skill testing, and rather move towards the integration of individuality among participants, in terms of traits. They suggest it is not only methodology in research that has to be revised, but also, and maybe more crucially, the mentality behind research strategies and intentions, as well the attitudes towards the applicability of the outcomes. In that scope, Digard and Sorace [56] emphasise the urge to improve the terminology used when conducting research where disorders are involved (they *provide the examples of “severity”, “affected”, “spared”, or “normal”) and shift towards a less “stigmatising” approach, the neurodiversity approach, that rely on promoting the variability as an alternative, rather than focusing on the impairments and lack of abilities [57].

A very interesting set of suggestions towards the inclusion of heterogeneity of autism and bilingualism, as well as the inclusivity of research methodologies has been demonstrated by Kašćelan and De Cat [58] in their review of “Bilingual language development in autism” by Prévost and Tuller [54]. The authors suggest that all four dimensions identified by Prévost and Tuller, namely ASD, cognition, impairment and bilingualism should be conceptualised as a continuum, aiming at a more empirical approach of the verge of their advantages and weaknesses (“constellation of continua approach”), which follows the same direction as the Research Domain Criteria Initiative (RDoC) [59]. The constellation of continua approach is promoted by the authors for embracing the obvious lack of homogeneity across the spectrum of autism in combination with the factor of bilingualism, providing an advantageous solution to misrepresentations of groups of bilinguals or autistic individuals, while being compared to the, as authors term it, homogeneous “super controls”. One more suggestion made by the authors with respect to the approach of constellation of continua is the fact that it should be accompanied by open science practices and cooperation among researchers, once more putting emphasis on the need for consensus among the members of the scientific community, as well as the availability and access policies with regard to methodological tools and strategies. Finally, one crucial point raised by the authors that concerns both autism and bilingualism research approaches is the significance of qualitative research, that promotes the examination and assessment of individual variation, as well as the systematic consideration of diversity in stimuli processing strategies, which drifts away from the “right vs. wrong” data analyses, but rather advocates the possibility of alternative interpretations and original semantic attributions (e.g., in the case of the “literal” interpretation of pragmatics by individuals with autism).

In the process of shedding some light on the methodological approaches that aim to shift the focus away from labelling, what should be considered is the clinical symptomatology of autism that is evident across the general population. Autism prevalence is reported to have expanded since the diagnostic criteria correspond to a much broader definition [60]. Autistic traits have been systematically detected among neurotypical undiagnosed individuals, a fact that has led to more inclusive assessments and diagnostic tools. Ruzich et al. [61] make a particular reference to the Autism-Spectrum-Quotient (AQ) as a self-report tool for evaluating general clinical and nonclinical populations. AQ is not only an assessment measure for autism, but also for a variety of neurodevelopmental disorders and clinical conditions, such as anorexia, schizophrenia, prosopagnosia, etc. The authors conducted a large-scale systematic literature review (including 73 articles) in order to detect research studies that were making use of this tool having non-clinical adult individuals as participants. Their meta-data reveal an average score of around 17 in the AQ with non-clinical individuals and around 35 with individuals with an autism diagnosis. Interpreting these findings, it can be seen that a certain amount of autistic traits are prevalent in the general population—to a lesser extent for the non-clinical populations in comparison to the clinical ones. The review provides a more well-founded base of interpretation of the AQ score, pointing out that this measure does not constitute a diagnostic tool but rather an evaluation of the emergence of autistic traits, both to diagnosed and undiagnosed populations [62].

Another crucial point to take into account is the developmental trajectory of autistic traits throughout time across the general population and whether they remain substantial or gradually fluctuate. In that scope, Robinson et al. [63] conducted a longitudinal study including parent and children assessment tools, over a time period of 6 years. The results reveal that autistic traits tend to present significant stability over time in the general population, both in low- and high-scoring groups. Performance with regard to the variable of sex demonstrated consistent stability in symptomatology as well. The findings suggest no fluctuation in terms of phenotypic autistic expectations over time, enhancing the continuity of the distribution of autistic traits. In terms of diagnostic relevance, the outcome of general population-based research studies supports the creation of categorisations among clinical, sub-clinical and non-clinical phenotypes within the spectrum of autism. These types of categorisations signify the need for not only better diagnostic tools, but also the design of prognostic measures [64].

Regarding studies that have explored the language and cognitive skills of children with ASD, it is well established that research findings are easily impacted by outliers. Given the intrinsically heterogeneous nature of behavioural performance in ASD and considering that atypical performance patterns across ASD individuals may be expected, choosing individual differences when assessing their language and cognitive profiles, rather than pre-specified diagnostic labels, is not trivial. Despite the fact that confounding effects of heterogeneity in ASD research have been highlighted in previous studies, individual differences have only recently gained attention in clinical and even nonclinical samples. Individual differences in studies with bilingual children and children with ASD should reveal fine-grained aspects of variability in the children’s cognitive and language performance by skipping the identification of a generalisable pattern that is often established through labelling.

Although working and creating indices is a novel approach that is gaining ground in the field of bilingualism, it is not found in studies that deal with autism. However, it is evident that the majority of the research studies in autism present specific limitations with respect to the individual variation of the symptomatology as well as factors such as sex, age, sample size, instrument of measuring language and cognitive abilities and generalisations of the research outcomes on the total of the population within the continuum of autism.

It is, therefore, evident that the practical shift towards the elimination of labels, particularly in the case of clinical populations, is not without problem. All things considered, methodological discussions should be raised, regarding transitional approaches with actual practical implementation in research. Such an approach could entail a method of profiling the populations under examination into less heterogeneous categorical subclusters that take into account a combination of cognitive, social, demographic and biological variables, as well as the comorbidity of different conditions with similar symptomatology [65]. However, this type or subgrouping should be accompanied by strategies of validation of the groups and replication of the findings [66]. In their systematic review of over 150 research articles that subgroup participants with autism, van Rentergem et al. [67] depict the lack of methodological homogeneity in the process of validation of outcomes and diagnostic subtypes. They propose validation techniques that are tailored to correspond to the desired outcomes, taking into consideration developmental and temporal trajectories. The authors analyse a variety of different validation techniques used in the research articles they reviewed and suggest a systematic validation checklist (Subtyping Validation Checklist—SUVAC) for inaugurating the creation of more solid subclusters of clinical populations, that will facilitate research replicability and outcome credibility.

The aforementioned methodological approaches put emphasis on the fact that simply incorporating interdisciplinary data occurring from multidimensional research studies on autism does not promote improvement in terms of replicability or methodological validity. The action that should be taken is towards a more equivalent subgrouping in terms of various variables (demographics, sex, age, severity of disorder), which will integrate the heterogeneity of autism and provide a more accurate categorisation of the individual differences on a larger scale [68].

## 5. Conclusions

This paper constitutes the first attempt to discuss the important issue of categorisation of individuals, by re-examining labels as well as placing emphasis on the role of individual differences. The number of research articles that indicate conflicting results is constantly growing. The lack of consensus with respect to a specific definition and criteria of bilingualism, in combination with the assimilation of any type of differentiation at the individual level once the labelling has been assigned (bilingual vs. monolingual, typical vs. atypical, etc.) reinforces the deviation and the non-replicability of the research findings. This matter should concern our field and cause us to question whether this labelling is valid at all. It is also very crucial to consider, when investigating spectrum-trait cases, such as bilingualism and autism, how heterogeneity should be defined and the extent to which it has an impact on the research. Heterogeneity in spectrum-trait cases might not be as diverse from one case to another as might have been considered so far [69]. It is a fact that the notion of variability has been addressed in research for quite some years now, placing emphasis on the significance of studying the individual profiles of participants in different domains of research and clinical practice [18,70,71], and the present study is in line with what these research investigations suggest. However, we are under the impression that even more awareness should be raised on that matter and that this methodological approach should demonstrate prevalence. We believe that more light should be shed on individual differences across all research studies, and we suggest that research should critically reconsider the view that individual variation is just another variable that should be included in the data. On the contrary, we further support the idea that we should usher research towards making it the essence of analysis.

## Figures and Tables

**Table 1:** Overview of the selected research studies with respect to individual variation in bilinguals.

Authors	Proposals
[14]	The authors propose that the field should move away from monolithic bilingual versus monolingual comparisons. Instead, they argue that research should focus on the experiences of the bilingual groups as predictors of structural changes in the brain, and also employ longitudinal designs to test the dynamic effects of active bilingualism.
[15]	She highlights the problem of labelling, and she proposes treating bilingual’s language experiences as a continuous variable.
[16]	The authors point out the problem of categorisation. According to them bilingualism should be considered a continuous variable.

**Table 2:** Overview of the selected research studies with respect to heterogeneity and individual variation in autism spectrum disorder.

Authors	Proposals
[7]	The authors suggest three points to take into consideration in autism research: they reject the notion of one single unifying diagnosis for autism that can be restrained under one neurobiological deficit. They point out that brain dysfunction and autistic symptomatology do not have a one-to-one analogy. They address the issue of data analysis methodology towards a more inclusive model regarding individual variation.
[39]	The authors suggest more fine-grained multidimensional and longitudinal approaches in autism research that focus on heterogeneity in terms of individual variation from an early age that has an impact on the holistic clinical image of individuals within the spectrum.
[40]	The authors provide an overview of the differences in the concept of autism in research and indicate the shift from categorical stratifications (autistic vs. non autistic) to the consideration of multidimensional diagnostic approaches and individual variation.
[41]	The authors take a holistic approach of investigation across the spectrum, emphasising on the heterogeneity of autism by exploring individual profiles of autistic children with respect to language and cognitive abilities.
